# Assessing Patient Satisfaction Following Facelifts with Social Media Reviews

**DOI:** 10.1007/s00266-024-04273-x

**Published:** 2024-08-01

**Authors:** Lucy Revercomb, Aman M. Patel, Hannaan S. Choudhry, Sadiq Shaikh, Christopher C. Tseng, Andrey Filimonov

**Affiliations:** 1 Department of Otolaryngology–Head and Neck Surgery, Rutgers New Jersey Medical School, Newark, NJ 07103 USA; 2 Department of Otolaryngology-Head and Neck Surgery, Pennsylvania State University College of Medicine, Hershey, PA 17033 USA

**Keywords:** Facelift, Social media, Patient satisfaction, Aesthetic outcomes

## Abstract

**Background:**

Facelifts are one of the most common facial aesthetic surgery procedures. Patient satisfaction determines success of most aesthetic surgery but has been historically difficult to assess.

**Objective:**

This study evaluated reviews by facelift patients on the aesthetic surgery social media website RealSelf.com to determine positive and negative factors underlying patient satisfaction following facelifts.

**Methods:**

Facelift reviews were gathered from RealSelf.com with an automated web crawler. Reviews were categorized as positive or negative and by the primary and secondary reasons for the positive or negative review. Patient “worth it” and star ratings, physician specialty, and cost of procedure were also collected.

**Results:**

A total of 2153 facelift reviews were collected. Overall, 1986 (92.24%) were positive and 167 (7.76%) were negative. The most common overall reasons for a positive review were aesthetic results (*n*=1571, 79.10%) and bedside manner (*n*=1488, 74.92%). The most common overall reasons for a negative review were outcome (*n*=137, 82.04%) and bedside manner (*n*=82, 49.10%). Most facelifts were performed by plastic surgeons (*n*=1796, 83.42%). The greatest 5-star rating percentages were seen for oral and maxillofacial surgeons (*n*=29, 93.55%), otolaryngologists (*n*=96, 92.31%), and plastic surgeons (*n*=1642, 91.43%). Of patients who provided a “worth it” rating, 1216 (91.91%) stated that their facelift was “worth it.”

**Conclusion:**

Overall patient sentiment toward facelifts was positive. The factors most commonly affecting a positive patient experience were bedside manner and aesthetic results. Negative patient reviews were primarily attributed to dissatisfaction with aesthetic outcomes. Social media serves as a valuable tool for evaluating patient satisfaction with aesthetic surgery.

**Level of Evidence IV:**

This journal requires that authors assign a level of evidence to each article. For a full description of these Evidence-Based Medicine ratings, please refer to the Table of Contents or the online Instructions to Authors www.springer.com/00266.

## Introduction

A significant growth in both quantity and quality of facial aesthetic surgery has been seen over the past several decades, driven by the premium that our society has placed on youthful appearance and the increasing societal acceptance of cosmetic surgery. Our faces have been brought to the forefront of daily life with selfies and virtual meetings on high-definition cameras, making many more attentive to the details and changes of their facial aesthetics. Facelifts are one of the most popular procedures used to combat the appearance of aging, with over 72,668 facelifts performed in the USA in 2022, up 8% from 2021 [1]. With increasing demand for these procedures comes increasing expectations for outcomes and safety, which have been met by improvements in surgical technique and treatment strategy [2–5]. However, assessing outcomes in aesthetic surgery, and accordingly the effect of these advancements in surgical technique, has been historically difficult [6–9]. Patient satisfaction is the predominant factor determining success of most facial aesthetic surgery. If a given surgeon is satisfied with his or her results, but the patients themselves are not similarly satisfied, then the intervention cannot totally be considered a success. Accordingly, the qualitative assessment of patients’ experiences, perspectives, and perception of their appearance in aesthetic surgery is especially important.

Social media has become an increasingly important aspect of plastic surgery, with both patients and surgeons utilizing social media platforms [10–12]. Facial aesthetic surgery patients desire accurate and reliable information with which to make informed decisions, and can utilize social media to learn about potential procedures and providers [13]. A survey of 500 patients at one plastic surgery clinic in 2015 found that 95% of patients used the internet and 45% of patients used social media to obtain information prior to consultation with a plastic surgeon, with social media strongly influencing 40% of those who used it in selecting a surgeon [11]. Accordingly, surgeons can benefit from the use of social media to enhance marketing efforts and increase patient volume. Surgeons must also beware of the potential impact of negative comments that can damage a surgeon’s reputation [14–16]. RealSelf (Seattle, WA) is a social media platform with over 100 million yearly users and 30,000 verified physicians designed for patient-patient and patient-physician interaction surrounding cosmetic procedures [17]. Patients post-physician reviews, ratings, before-and-after photographs, and other information such as cost and location can be analyzed to ascertain the patient perspective on their experience.

The purpose of this study was to (1) evaluate patient satisfaction following facelifts and the underlying positive and negative factors that drive overall satisfaction based on social media reviews and (2) to characterize additional aspects, including physician specialty and cost, in the context of the facelift patient experience.

## Methods

Reviews posted under the topic “Facelift” on RealSelf.com were gathered with the automated Python-based web crawler Scrapy (White Plains, NY) from January 2019 to December 2021. Variables collected from each review included the review title, review category, review date, physician specialty, patient-reported “worth it,” the number of pictures associated with the review, and cost were extracted. To facilitate comparison between specialties, physician specialties were categorized as plastic surgery, otolaryngology, oral and maxillofacial surgery, ophthalmology, and other. The ratings provided by patients in different categories on a scale of 1 to 5 were also collected. These categories included overall rating, “doctor’s bedside manner,” “answered my questions,” “after care follow-up,” “time spent with me,” “phone or email responsiveness,” “staff professionalism and courtesy,” “payment process,” and “wait times.”

Each review was individually evaluated by the authors and categorized as either a positive or negative review. The reviews were then further classified into categories describing the main reason for the review. When reviews included secondary reasoning for being a positive or negative review, these additional secondary reasons were counted as secondary categories. Accordingly for each category a primary percentage was calculated as the proportion of positive or negative reviews for which the category was the primary reason, and a total percentage was calculated as the proportion of positive or negative reviews for which the category was the primary or secondary reason. An overall percentage was also calculated as the proportion of total reviews, both positive and negative, for which the category was the primary or secondary reason. No neutral category was included as our analysis suggested there were very few reviews that could be defined as neutral because all reviews generally expressed some sentiment regarding the patient facelift experience. Additionally, determining if a review was truly neutral, rather than positive or negative, likely would have increased subjectivity of this analysis.

Positive categories included aesthetic result, bedside manner, comfort, cost, and office. Aesthetic result included having a natural appearance, stitches not being too prominent, and minimal scarring. Reviews categorized as bedside manner included references to the surgeon being personable, a good listener, spending adequate time with the patient, and clearly explaining the procedure. Comfort included manageable postoperative pain levels. Office included pleasant support staff, short wait times, and an aesthetically pleasing office. Negative categories included appearance/outcome, bedside manner, cost, and logistics. Appearance/outcome included prominent stitches or scarring, worsening of appearance, unnatural appearance, persistent skin laxity, and asymmetry. Negative bedside manner included overconfidence, lack of empathy, dismissal of patient concerns, and lack of adequate time spent with the patient by the surgeon. Logistics included negative office experiences such as long wait times and uncomfortable surgery centers. This study was exempt from Institutional Review Board review because of the public, deidentified nature of the patient data.

## Results

A total of 2153 facelift reviews were collected from RealSelf.com and analyzed. Reviews were categorized by the patient’s expressed overall positive or negative sentiment regarding their experience (Table 1). Of the 2153 total reviews, 1986 (92.24%) reviews were positive and 167 (7.76%) were negative. Among the positive reviews, the most frequently cited primary reasons were bedside manner (*n*= 989, 49.80%) and aesthetic result (*n*= 950, 47.83%). Of the overall reasons cited for a positive review, aesthetic results (*n*= 1571, 79.10%), bedside manner (*n*= 1488, 74.92%), and comfort (*n*= 152, 7.65%) were found to be the most common. The most frequently cited primary reasons for negative reviews were appearance or outcome of the procedure (*n*= 120, 71.86%) and bedside manner (*n*= 35, 20.96%). Of the overall reasons cited for a negative review, appearance or outcome (*n*= 137, 82.04%), bedside manner (*n*= 82, 49.10%), and cost (*n*= 25, 14.97%) were the most common.

**Table 1:** Positive and negative review categories

Category	Primary reason (n, %)	Secondary reason (n, %)	Total primary and secondary reason (n, %)	Overall (n, %)
Positive	1986			1986 (92.24%)
Aesthetic result	950 (47.83%)	621 (48.40%)	1571 (79.10%)	1571 (72.97%)
Bedside manner	989 (49.80%)	499 (38.89%)	1488 (74.92%)	1488 (69.11%)
Comfort	31 (1.56%)	121 (9.43%)	152 (7.65%)	152 (7.06%)
Cost	5 (0.53%)	15 (1.17%)	20 (1.01%)	20 (0.93%)
Office	11 (0.55%)	27 (2.10%)	38 (1.91%)	38 (1.76%)
Negative	167			167 (7.76%)
Appearance/outcome	120 (71.86%)	17 (20.00%)	137 (82.04%)	137 (6.36%)
Bedside manner	35 (20.96%)	47 (55.29%)	82 (49.10%)	82 (3.81%)
Cost	8 (4.79%)	17 (20.00%)	25 (14.97%)	25 (1.16%)
Logistics	4 (2.40%)	4 (4.71%)	8 (4.79%)	8 (0.37%)
Total	2153			

Patient review ratings evaluated several metrics, with 1 star corresponding to the lowest rating and 5 stars corresponding to the highest possible rating (Table 2). Overall, the overwhelming majority of patients (*n*= 1920, 93.02%) gave the highest overall rating of 5 stars for their doctor. Beyond overall rating, the most frequently cited reasons for a 5 out of 5 rating were “doctor’s bedside manner” (*n*= 397), “staff professionalism and courtesy” (*n*= 396), “payment process” (*n*= 394), and “answered my questions” (*n*= 393). The most frequently cited reasons for the poorest rating of 1 out of 5 were “after care follow-up” (*n*= 23), “doctor’s bedside manner” (*n*= 14), “phone or email responsiveness (*n*= 13), and “payment process” (*n*= 13).

**Table 2:** Five-star review categories and ratings

Category	Rating (number of stars)					
	1	2	3	4	5	N/A
Overall rating	104 (5.04%)	20 (0.97%)	9 (0.44%)	11 (0.53%)	1920 (93.02%)	89
Doctor's bedside manner rating	14 (3.26%)	4 (0.93%)	5 (1.17%)	9 (2.10%)	397 (92.54%)	1724
Answered my questions rating	11 (2.57%)	8 (1.87%)	9 (2.10%)	7 (1.64%)	393 (91.82%)	1725
After care follow-up rating	23 (5.39%)	6 (1.41%)	3 (0.70%)	13 (3.04%)	382 (89.46%)	1726
Time spent with me rating	11 (2.60%)	7 (1.65%)	7 (1.65%)	14 (3.31%)	384 (90.78%)	1730
Phone or email responsiveness rating	13 (3.06%)	5 (1.18%)	6 (1.41%)	17 (4.00%)	384 (90.35%)	1728
Staff professionalism & courtesy rating	12 (2.81%)	7 (1.64%)	4 (0.94%)	8 (1.87%)	396 (92.74%)	1726
Payment process rating	13 (3.07%)	3 (0.71%)	3 (0.71%)	10 (2.36%)	394 (93.14%)	1730
Wait times rating	7 (1.65%)	3 (0.71%)	11 (2.59%)	28 (6.59%)	376 (88.47%)	1728

N/A, not available

Most facelifts in our study cohort were performed by plastic surgeons (*n*= 1796, 83.42%), followed by other or non-specified specialties (*n*= 162, 7.52%), and otolaryngologists (*n*= 104, 4.83%) **(**Table 3**)**. Facelifts were also performed by ophthalmologists (*n*= 60, 2.79%) and oral and maxillofacial surgeons (*n*= 31, 1.44%). The specialties with the greatest proportion of 5-star ratings were oral and maxillofacial surgeons (*n*= 29, 93.55%), otolaryngologists (*n*= 96, 92.31%), and plastic surgeons (*n*= 1642, 91.43%). The lowest proportion of 5-star ratings were seen among ophthalmologists (*n*= 53, 88.33%) and other or non-specified specialties (*n*= 100, 61.73%).

**Table 3:** Physician specialties associated with 5-star ratings

Physician specialty	Number of reviews (n, %)	Number of 5-star ratings (n, %)
Otolaryngology	104 (4.83%)	96 (92.31%)
Plastic surgery	1796 (83.42%)	1642 (91.43%)
Oral maxillofacial surgery	31 (1.44%)	29 (93.55%)
Ophthalmology	60 (2.79%)	53 (88.33%)
Other	162 (7.52%)	100 (61.73%)

A total of 1323 reviews provided a “worth it” rating. Among these reviews, 1216 (91.91%) patients stated that their facelift was “worth it,” 81 (6.12%) patients stated that their facelift was “not worth it,” with the remaining 26 (1.97%) stating “not sure” (Table 4). Photographs taken during the preoperative and/or postoperative stages were provided in 1897 reviews (88.11%). A total of 301 reviews (13.98%) provided the cost of their facelift. Among these reviews, the average facelift cost was $15,247.71, ranging from $1.00–$75,000.00.

**Table 4:** Additional review statistics

Variable	Frequency (n, %)
*Patient Worth It*	
Worth it	1216 (91.91%)
Not worth it	81 (6.12%)
Not sure	26 (1.97%)
N/A	830
*Photographs provided*	
Yes	1897 (88.11%)
No	256 (11.89%)
*Cost*	
Cost provided	301 (13.98%)
No cost provided	1852 (86.02%)
Mean ($)	$15,247.71
Median ($)	$13,000.00
Minimum ($)	$1.00
Maximum ($)	$75,000.00
*Years included*	
2019	525 (28.46%)
2020	643 (34.85%)
2021	677 (36.69%)

N/A, not available.

## Discussion

The most importance outcome for cosmetic procedures is patient satisfaction which is influenced by a variety of factors including aesthetic outcomes, interactions with the physician and staff throughout the experience, and the postoperative recovery course. A few validated outcome measures exist for facelifts, including the Facelift Outcomes Evaluation, Face-Q, and Owsley Facelift Outcomes Evaluation; however, they are not conventionally used in the literature, making it difficult to evaluate outcomes of various techniques [4, 9, 18, 19]. Social media use for both patients and physicians has become prominent throughout the field of cosmetic surgery. Evaluation of qualitative data from social media reviews can provide unique insight into the patient perspective, which may not be obtained from surgeon-initiated patient surveys where patients may be hesitant to express their dissatisfaction. This study examined reviews from RealSelf.com, which provides a large sample of patient reviews detailing the patient perspective, to further understand the factors associated with patient satisfaction following facelifts.

Our study demonstrated an overall positive patient sentiment toward their facelift. Rates of patient satisfaction were consistent across the various measures utilized, with 92.24% of reviews categorized as positive, 91.91% of patients deeming their facelift “worth it,” and 93.02% of patients giving their physician an overall five out of five-star rating. Previous literature evaluating patient perspectives on facelift outcomes through surveys such as the Facelift Outcomes Evaluation, the Owsley Facelift Satisfaction Survey, and the Face-Q, reported similar findings of 85.9%, 92.1%, and 90.5% patient postoperative satisfaction, respectively [19–21]. Satisfaction with facelifts as measured by percent “worth it” is comparable to satisfaction with blepharoplasty and rhinoplasty which were reported as “worth it” by 93.5% and 93.8% of patients on RealSelf, respectively [10, 22]. A similar study performed for breast reconstruction reported a 89.4% “worth it” rate by patients [23].

In our study, the most frequent primary reason for a positive facelift experience was surgeon bedside manner (49.80%) and aesthetic result (47.83%). The patient emphasis on bedside manner aligns with studies throughout the literature demonstrating the relationship between patient satisfaction with their procedure and their perception of their care provider and the degree to which their needs were met [6, 24, 25]. To improve patient satisfaction with facelifts, physicians must aim not only for high aesthetic outcomes but also to earn the patient’s trust and confidence and leave them feeling well-cared for throughout the clinical encounter. Aesthetic result, as the second most common primary reason and the most common overall cited reason for a positive review, was also very important to patients. A previous evaluation of facelifts and necklifts on RealSelf established that the most frequent reason patients pursued the procedure was due to their face looking older/aged/wrinkled/sagging and that the main reason for being satisfied with the surgical outcome was looking younger and fresher [26]. The importance of the aesthetic result is also demonstrated in that the most referenced reason for a negative review by far was a poor aesthetic appearance or outcome (71.86%). The distribution of positive and negative reviews attributed to bedside manner and aesthetic outcomes potentially reflects the expectation by patients of a positive aesthetic result, and the according disappointment if they are not satisfied with their results, and the enhancement of their experience if they are additionally pleased by the physician interaction.

In addition to “worth it” ratings, patients on RealSelf are able to rate specific categories detailing their experience on a scale from one to five stars. The proportion of 5-star reviews (93.02%) coincided with the proportion of positive reviews established by author categorization and “worth it” ratings. Secondary ratings were also overwhelmingly positive, with most being greater than 90% 5-star. The only secondary ratings below 90% were for after care follow-up (89.46%) and wait times (88.47%), with prolonged wait times being a known common complaint for patient clinical encounters [6, 27]. Only 14% of reviews provided the cost of the procedure, and the mean of $15,247.71 for given costs from our sample of reviews is higher than the average cost of $9,281 reported by the American Society of Plastic Surgeons for facelifts in 2022 [1]. This may be attributed to the small sample size of reviews that reported cost and the minority of positive reviews addressing cost (1.01% of positive reviews) compared to the proportion of negative reviews addressing cost (14.97% of negative reviews). The disproportion of positive and negative reviews regarding cost may suggest that patients upset by greater costs for their procedure more frequently reported the cost in their review.

Plastic surgeons performed the majority of facelifts in our study cohort (83.42%). This is consistent with previous studies showing plastic surgery performing the most outpatient cosmetic procedures, including facelifts (62.40%) [28, 29]. Although oral maxillofacial surgeons had the highest overall patient satisfaction rating with 93.55% of their reviews having 5-star ratings, there were very few instances of oral maxillofacial surgeons (*n* = 31) performing a facelift, and thus, it was difficult to ascertain the significance of this finding. The percentage of 5-star ratings was also fairly similar to otolaryngologists (92.31%), plastic surgeons (91.43%), and ophthalmologists (88.33%). Specialties outside of plastic surgery, otolaryngology, ophthalmology, and oral maxillofacial surgery, including dermatology and unspecified specialties, had a much lower overall patient satisfaction of only 61.73%. These findings highlight the benefit of expertise and experience with facelifts in achieving high patient satisfaction.

This study has several notable limitations. There is the potential overrepresentation of decidedly positive and negative patient reviews due to the tendency of those with polarizing opinions to be especially motivated to share their experience on social media. There also may be overrepresentation of those more comfortable using the internet and social media. Although patient evaluation of their own facelifts allows for assessment of patient satisfaction, there are inherent limitations of patients subjectively analyzing their own facelifts. This may include pre-existing biases about their appearance leading to positive or negative self-perception, comparison to unrealistic expectations, and emphasis on positive or negative aspects to justify or regret their decision. It is also possible for physicians to target patients with positive outcomes and encourage them to post online, potentially biasing their results. However, RealSelf independently verifies each review’s authenticity and assesses for potential conflicts of interest before the reviews are published and prohibits physicians from unilaterally eliminating negative reviews [30]. This study was also limited by RealSelf’s characterization of all facelifts under the topic “facelifts” and the lack of inclusion of the specific type of facelift in many reviews. This limited the ability to distinguish between different techniques including soft-tissue techniques, implants or adjunctive medications, and adjunctive techniques such as fat grafting, resurfacing, and liposuction, which have varying levels of invasiveness, indications, and postoperative outcomes [3, 4]. Reviews also varied in detail, length, and quality of information provided and the subjectivity of each review limits the generalization of our findings to the overall facelift population. Despite these limitations, the large sample of facelift reviews on RealSelf and the data provided are valuable in ascertaining the patient perspective on facelifts and what factors influence the satisfaction with their procedure.

## Conclusions

Our study utilized patient reviews posted on RealSelf.com to identify factors associated with positive and negative facelift experiences. The aesthetic outcome was one of the most common primary reasons for both positive and negative reviews, emphasizing the importance of the patient’s own satisfaction with their appearance following cosmetic procedures. The patient-physician relationship was also a key factor in patient satisfaction, with positive bedside manner frequently addressed in patient reviews. Social media serves as a valuable tool for garnering patient perspective on procedures and can provide beneficial information to both prospective patients and physicians aiming to improve patient satisfaction and outcomes.
